# Effective Elastic Modulus of Structured Adhesives: From Biology to Biomimetics

**DOI:** 10.3390/biomimetics2030010

**Published:** 2017-06-29

**Authors:** Xin Wang, Di Tan, Xinyu Zhang, Yifeng Lei, Longjian Xue

**Affiliations:** School of Power and Mechanical Engineering, Wuhan University, South Donghu Road 8, Wuhan 430072, China; yikaleide@whu.edu.cn (X.W.); Di-Tan@whu.edu.cn (D.T.); kidow@foxmail.com (X.Z.); 00031503@whu.edu.cn (Y.L.)

**Keywords:** effective elastic modulus, hierarchical structure, adhesion, self-similar structure, lamella–pillar hybrid structure, porous structure

## Abstract

Micro- and nano-hierarchical structures (lamellae, setae, branches, and spatulae) on the toe pads of many animals play key roles for generating strong but reversible adhesion for locomotion. The hierarchical structure possesses significantly reduced, effective elastic modulus (*E_eff_*), as compared to the inherent elastic modulus (*E_inh_*) of the corresponding biological material (and therefore contributes to a better compliance with the counterpart surface). Learning from nature, three types of hierarchical structures (namely self-similar pillar structure, lamella–pillar hybrid structure, and porous structure) have been developed and investigated.

## 1. Introduction

Several groups of animals (gecko, beetle, spider, fly, and tree frog among others) possess outstanding locomotive abilities on various surfaces in different environments. Exquisite micro- or nano-hierarchical structures on their toe pads, which are normally divided into hairy (setal) and smooth pads, play a critical role in adhesion and are referred to as structured adhesives [1,2,3,4,5,6]. While the material for the conventional adhesive (e.g., pressure sensitive adhesives) normally possesses an inherent elastic modulus (*E_inh_*) lower than the Dahlquist criterion for tack (~100 kPa) [7,8], the *E_inh_* of the material for structured adhesives is far higher than the upper limit of the criterion [9,10]. Taking the tokay gecko (*Gekko gecko*) as an example, its setae are composed of a hard material (β-keratin) with an *E_inh_* of 2–4 GPa [10,11]. Considering the geometry of the hierarchical setal array, however, the gecko’s toe pads can rapidly form reliable contacts on surfaces with a roughness of different length scales [12,13]. The proper contact between the seta and the counterpart surface generates Van der Waals forces. Millions of these contact points per toe pad acquire enough adhesion force to support the body weight of the gecko [14,15]. In another example, the material at the tip of adhesive tarsal setae of the ladybird beetle (*Coccinella septempunctata*) is also composed of hard material (resilin) with an *E_inh_* ~7.2 GPa in the dehydrated state [16]. The materials with such high elastic moduli can hardly form effective contact with a rough surface (they can maybe only make a few contact points). Relying on the hierarchical structure on their toe pads, however, these animals can very well stick to, walk, run, and jump on various surfaces. In this case, the *E_inh_* of a material in bulk state is no longer appropriate to describe the structured material. Unlike the adhesive setal array on the gecko’s toe pad, the apparent elastic modulus (also referred to as effective elastic modulus, *E_eff_*) is widely used for structured materials.

**Figure 1:**
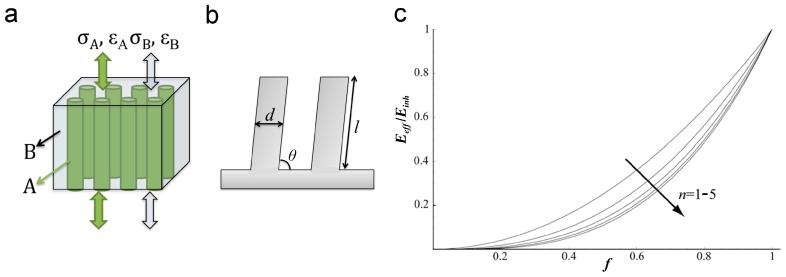
Proposed models to describe a pillar array. (**a**) A composite model of aligned fibers/pillars (A, green) embedded within a matrix (B gray) shows the strain (*ε*) under the stress (*σ*) along the long axis of fibers/pillars; (**b**) Schematic of a pillar array with pillar diameter *d*, length *l* and tilting angle *θ*; (**c**) Dependence of the effective elastic modulus (*E_eff_*) of the structured adhesive normalized to inherent elastic modulus (*E_inh_*) expressed as a function of the filling ratio (*f*) for hierarchical levels *n* = 1–5. Reproduced with permission from [22].

Both hairy and smooth pads can be considered as pillar arrays, but with different aspect ratios (ARs). The *E_eff_* of biological structured adhesives can be estimated using a modeled composite that consists of aligned fibers/pillars embedded within a matrix (Figure 1). The fibers/pillars and the matrix are defined as A and B, and their inherent elastic moduli are *E_A_* and *E_B_*, respectively. Assuming that the model composite has strong interfacial interactions between the fibers/pillars and the matrix, and deforms elastically under the force along the long axis of aligned fibers/pillars (Figure 1a), Hooke’s law can be used to describe the deformation of the composite. The same strain occurs in the two components (*ε* = *ε_A_* = *ε_B_*), and the *E_eff_* of the model composite can be given by:

(1) $$E_{eff}=E_{A}V_{A}+E_{B}V_{B}$$

where *V_A_* and *V_B_* = 1 − *V_A_* are the volume fractions of A and B, respectively. For the smooth pad on animals such as the tree frog, the polygonal pillar is the component A, and the adhesive secretion in the microchannels between the pillars is the component B. For the dry hairy pads found in geckos and spiders, the matrix B is air; the *E_eff_* of these hairy adhesives could then be roughly estimated from the volume fraction of component A:

(2) $$E_{eff}\to E_{A}V_{A}$$

Since *V_A_* is always smaller than 1, the *E_eff_* of the composite is smaller than *E_A_*. A lower *E_inh_* means a larger deformability of the material. Therefore, the structured material has higher elastic energy dissipation [17,18] and higher possibility to generate more contact points on the counterpart surface, especially on the surface with a certain roughness [19,20].

More specifically, if the component A forms the ordered array of pillars (Figure 1b) with pillar diameter *d*, pillar length *l*, tilting angle *θ* with respect to the supporting layer (0° < *θ* ≤ 90°), and pillar density *ρ*, the *E_eff_* of such pillar array can be estimated from the following equation [10,21]:

(3) $$E_{eff}=\frac{3\pi E\rho d^{2}\cos\left(\theta \right)}{64{\left(\mathrm{AR}\right)}^{2}\sin^{2}\left(\theta \right)}$$

where the aspect ratio (AR) of the pillar is defined as the ratio between the pillar length *l* and its diameter *d*. The increase in the AR of the pillar, which could be realized by increasing *l* or reducing *d*, or combining both, reduces the *E_eff_* of the pillar array. Long pillars and pillars with a smaller diameter have a greater chance to form effective contacts on a rough surface, even with the valley part of the roughness [21]. However, the increase of the AR will reduce the stability of the pillar array. To balance these two aspects, biological systems normally adopt a hierarchical design composed of structures over several length scales. Taking into account the hierarchy and the tilted configuration of pillar arrays, Schargott [22] proposed a three-dimensional (3D) model to describe the *E_eff_* of such hierarchical systems. The *E_eff_* decreases by reducing the filling ratio *f* of the pillars, or in other words, by increasing the space among pillars. Moreover, *E_eff_* can be further reduced by the introduction of more levels of hierarchy. Therefore, the pillar array with more levels of hierarchy (*n* = 1–5 in Figure 1c) could gain better adhesion performance. The 3D model also indicates that the increase in the roughness of the counterpart surface reduces the adhesion performances for the pillar arrays with any hierarchical levels, which is caused by the reduction of contact possibilities. 

The dependence of adhesion on the contact possibility could be evaluated by the spring model [23,24]. According to this model, each pillar is considered as an independent spring with a spring constant *E*/*Al*_0_, where *E* is the elastic modulus of the pillar material, *l*_0_ is the original length of the pillar, and *A* is the contact area between the pillar tip and the counterpart surface. It assumes that the probability *p* of a pillar forming contact with the counterpart surface is linearly proportional to the indentation depth Δ*l* until *p* reaches 100%, and the contact persists until the pull-off. Larger loading force causes more contact points and generates a larger adhesion force. Mimicking the hierarchical adhesive structures, the multilevel spring model was proposed to evaluate the influence of hierarchy on the adhesion performance. It suggests that the hierarchical pillar array with more levels, smaller elastic modulus, and larger preload possesses better adaptation to rough surfaces, enhancing adhesion significantly [25,26]. The setting of stiffness is to be 1/10 of the typical value of the setae of tokay gecko, and the root mean square (RMS) roughness of the counterpart surface is to be 3 μm, resulting in an adhesion coefficient (defined as the ratio of adhesion force to loading force) of 260% in the three-level structure, which is much higher than that of the one-level structure [27]. 

Besides the above-mentioned basic structures, other structural parameters like tip geometry, tilt angle, density, and composition variety also contribute to the overall *E_eff_* of the structured adhesives [10,22]. In the following sections, we will discuss the influence of these structural parameters on natural and artificial structured adhesives.

## 2. Naturally Occurring Hierarchical Structured Adhesives

**Figure 2:**
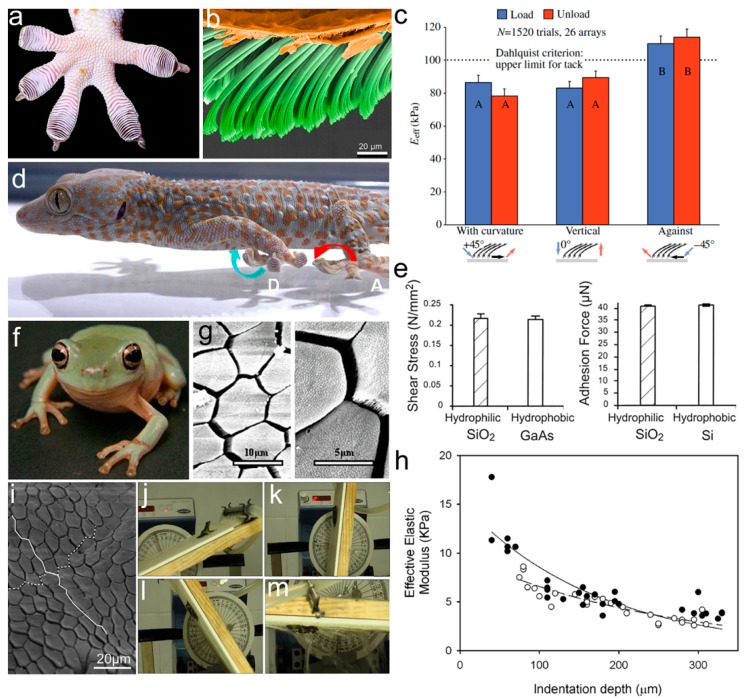
Hierarchical structured adhesives in nature. (**a**) Toe pad of tokay gecko. Reproduced with permission from [10]; (**b**) Scanning electron microscopy (SEM) image of gecko’s toe pad hierarchical structure of multi-level setae (green), supported by lamella (orange). Adapted with permission from [12]; (**c**) Effective elastic modulus (*E_eff_*) during deformation of isolated setal arrays on the gecko’s toe pad. Reproduced with permission from [10]; (**d**) Attachment (A) and detachment (D) processes of the gecko’s toe pad. Reproduced with permission from [40]; (**e**) The performance of gecko setae on hydrophilic SiO_2_ and hydrophobic GaAs or Si surfaces. Reproduced with permission from [14]; (**f**) Immature White’s tree frog (*Litoria caerulea*); (**g**) SEM images of the toe pad of White’s tree frog at different magnifications. Reproduced with permission from [55]; (**h**) Effect of indentation depth on the effective elastic modulus of the tree frog smooth adhesive pad during indentation test. Open and filled circles represent data from two mature adult frogs. Reproduced with permission from [60]; (**i**) Elongated polygonal epithelial cells on the toe pad of the torrent frog (*Staurois guttatus*); (**j**–**m**) Adhesion performance of *Staurois guttatus* on the rotating platform, with an uneven surface under high flow velocity conditions. Reproduced with permission from [65].

To ensure reliable adhesion in complex natural environments, animals have evolved elaborate hierarchical adhesive structures on their toe pads. As a typical example with the most sophisticated structure, the multilevel setal array on the toe pads of the tokay gecko has been widely studied [28,29,30,31]. The first level of hierarchy is 15–20 slices of lamellae, stretched from the toe pad (Figure 2a). Each lamella is covered with a large number of primary setae (30–130 μm in length, 5–10 μm in diameter, and ~14,000 setae/mm^2^ in density), tilting at a certain angle towards the tarsal end of the toe pad (the second level, Figure 2b). Some secondary setae with a length of 2–3 μm and a diameter of 200–300 nm (the third level) split from the primary seta, while the spatular tip could be considered as the fourth level. The unique hierarchical structure on the gecko’s toe pads offers the setae array an *E_eff_* of 83 ± 4.0 kPa, well below the Dahlquist criterion for tack [10]. A small *E_eff_* allows the setae array to acquire high compliance to rough surfaces, forming a large number of contact points on the touching surface [32]. The adhesion tests of live geckos on rough surfaces revealed a dependence of gecko adhesion on the surface roughness, relative to the hierarchical structure on the gecko’s toe pads. Gecko adhesion was found to decrease on the surface, with amplitudes and wavelength approaching the size and the interspace of lamellae [33], setae [34], and spatular tips [35,36,37,38]. For example, examination of gecko adhesion revealed a 95% reduction of shear adhesion on the engineered substrate (constructed with sinusoidal patterns, with amplitudes and wavelengths in sizes similar to the dimensions close to the lamella length, and interlamellar spacing) [33]. However, on sinusoidal surfaces with amplitudes much larger than the gecko setae, spatular tips can increase adhesive forces by 2.5 times on smooth surfaces and 10 times on rough surfaces [39]. 

Interestingly, an *E_eff_* of the setae array less than 100 kPa can only be detected when a shear force is applied in the direction of the natural curvature of the setae array or a normal loading force is applied on its surface (left two columns indicated with A in Figure 2c). However, an *E_eff_* larger than 100 kPa can be detected when a force is sheared against the setal curvature (right column indicated with B in Figure 2c). The shear force-dependent *E_eff_* thus offers an easy way to control the adhesion/friction performance. It explains why the gecko rolls up its toe, starting from the tarsal end (against the setal curvature) during detachment (blue arrow in Figure 2d) [15,40]. 

The fine hierarchical structure of setae found on the gecko’s toe pads allows enough contact points with natural surfaces to collect enough adhesion for locomotion [29]. Based on the finding that gecko adhesion is more or less the same on hydrophilic and hydrophobic surfaces, Van der Waals forces (rather than capillary forces) were suggested to dominate gecko adhesion (Figure 2e) [14]. On the other hand, however, gecko adhesion increases in humid environments (with a humidity range between 30 and 80% [41,42]); even a ~68% decrease in shear adhesive force was detected on the toe pad when stepping on a wet hydrophilic surface [43]. The humidity-enhanced gecko adhesion is rationalized by the softening of setal keratin in humid environments [44]. In addition, electrostatic forces [45] and vacuum pressure [46,47] are also suggested to contribute to gecko adhesion.

Analogous to the setal array of the gecko, a smooth adhesive pad can be considered as a pillar array with low AR [48,49,50]. Taking the tree frog (*Litoria caerulea*) as an example (Figure 2f) [51,52,53], its toe pad consists of numerous polygonal epithelial cells (the first level of hierarchy) with a diameter of ~20 μm, separated by microchannels with 2–3 μm in width and ~5 μm in depth. A single polygon (mostly a pentagon or hexagon) is composed of a dense array of nanopillars (e.g., keratin nanopillars on the toe pad of the rock frog, *Staurois parvus*), which are the second level (Figure 2g) [54,55]. The polygonal hierarchical structures increase adhesion/friction 2–3 times on the surface with small-scale roughness (3–6 μm asperities), and show relatively poor adhesion on the surface (with tested roughness asperities ranging from 58.5 to 562.5 μm) [56,57,58]. Furthermore, the polygonal structure has a constituent gradient from top to bottom, due to the existence of a dense network of capillaries beneath the pad epidermis [55,59]. For example, the elastic modulus of the keratinized layer on the toe surface of the tree frog was detected to be 5–15 MPa, and the *E_eff_* continued to decrease with the increase of the indentation depth (Figure 2h) [60]. This unique gradient structure is believed to have the function of keeping good abrasion resistance, meanwhile improving adhesion abilities on uneven surfaces. Not only in smooth pads the constituent gradient was also found in the fresh tarsal seta of the ladybird beetle [16]. Two significant longitudinal gradients in the material composition, together with the hydration state, generate a tremendous change of elastic modulus from the setal tip (1.2 ± 0.3 MPa) to the base (6.8 ± 1.2 GPa) in the fresh seta. The huge gradient in modulus can simultaneously offer the seta a high stability and a high compliance to the counterpart surface during the locomotion process. However, the gradient is lost in a dried seta and the modulus increases to ~7.2 GPa. It infers the critical role of water in determining the *E_eff_* of the seta, and in keeping the biological tissue function. The modulus gradient can also be considered as a hierarchical structure, enhancing adhesive capacity and structural stability at the same time.

On the smooth adhesive pads of frogs, mucus secreted from mucous glands spreads over the surface of the pad through microchannels forming a continuous thin liquid film. This liquid film contributes to contact formation and thus frog adhesion is referred to as wet adhesion [61,62] compared to dry adhesion in geckos. However, the mechanism for wet adhesion is too complex to be precisely described at present. On one hand, the liquid at the contact interface introduces capillary and hydrodynamic forces [63,64]; on the other hand, the mucous film may help to squeeze water out from the contact interface, generating direct contacts [65]. The squeezing effect is suggested to be strongly influenced by the pattern of microchannels, which may be responsible for the adaption of various species to different habitats. In contrast to the hexagonal patterns on the toe pads of tree frogs (Figure 2g), elongated polygonal epithelial cells (together with straight channels along the distal–proximal axis (white line in Figure 2i) of the toe pads of torrent frogs (e.g., *Odorrana hosii* and *Staurois guttatus*) that live around streams and falls [65,66] are suggested to perform better at liquid draining. This highly specialized microstructure on the toe pad of the torrent frog can therefore provide larger adhesion and friction forces in fast-flowing water [53,65]. For instance, the torrent frog *Staurois guttatus* can keep attaching to a rotating coarse platform coated with 1125 μm-diameter particles (which simulates the conditions of its natural environment) from 0° to ~180° in fast flowing water (~4000 mL/min) (Figure 2j–m).

## 3. Bioinspired Hierarchical Structured Adhesives

Based on the understanding of remarkable structured adhesives in nature, synthetic structured adhesives have been developed and studied in detail in the last decade [67,68,69,70,71,72,73]. The hierarchical structure, rather than the surface chemistry of the toe pads of these animals, contributes mainly to the adhesion performance [1]. The hierarchical design offers the synthetic material significantly reduced *E_eff_*, and therefore a higher possibility to form more contact points with the counterpart surface, enhancing its adhesion properties. In the following sections, we discuss the influence of structure parameters on adhesion by dividing the hierarchical structures into three groups: self-similar hierarchical pillar, lamella–pillar hybrid structure, and porous structure.

### 3.1. Self-Similar Pillar Structure

#### 3.1.1. Self-Similar Hierarchical Pillar

The self-similar hierarchical pillar is defined as the pillar array with similar pillar structures and different dimensions on each hierarchical level. For example, on the gecko toe pad, the primary seta and the secondary setae have similar geometry but different sizes. In order to gain high adhesion by using the pillar array, a low *E_inh_* of the material (or a low *E_eff_* of the structure) and a high density of contact points are normally required [74,75]. The *E_eff_* of a pillar array can be effectively reduced by shrinking the pillar diameter or increasing the pillar AR. However, a single-level pillar tends to buckle/collapse, while these two requirements are achieved at the same time. Grafting smaller pillars (the second level) to the end of a larger pillar (the first level); in other words, forming a self-similar hierarchical pillar could fit the two prerequisites at the same time. Because of its smaller size, the density of the second-level pillar is much higher than that of the first level. However, the space among the second-level pillars, together with the space among the first-level pillars, contributes to a great reduction in the *E_eff_* of the second-level pillar array [76,77]. The array of self-similar hierarchical pillars thus possesses a lower *E_eff_*. The introduction of more hierarchical levels could be used to further reduce the *E_eff_*, in order to gain stronger adhesion [22].

**Figure 3:**
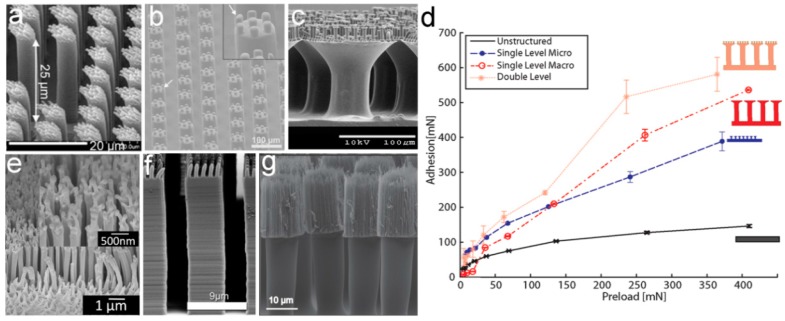
Two-level self-similar pillar arrays fabricated by various techniques. (**a**) Two-step molding with UV-curable polyurethane acrylate (PUA) resin. Reproduced with permission from [79]; (**b**) Polydimethylsiloxane (PDMS) replicated from a mold, prepared by two-step photolithography. Reproduced with permission from [80]; (**c**) Inking technique with polyurethane (PU); (**d**) Dependence of adhesion on preload for the unstructured, single primary structure (macro), single secondary structure (micro), and two-level structure. Reproduced with permission from [82]; (**e**) Capillary force-assisted molding from a multi-branched AAO template with grade Lexan polycarbonate (PC). Reprinted with permission from [86]. Copyright (2011) American Chemical Society; (**f**) Three-dimensional (3D) direct laser writing with acrylic-based negative photoresist (IP-G 780). Reproduced with permission from [89]; (**g**) Imprinting with carbon nanotube forests (CNTFs). Reproduced with permission from [90].

In order to gain adhesion abilities comparable to natural structured adhesives, many approaches have been carried out to prepare two-level self-similar pillar arrays [78]. Using the UV-curable polyurethane acrylate (PUA, *E_inh_* ~ 19.8 MPa), hierarchical pillars composed of slanted nanopillars (with spatula ends atop micropillars) have been acquired by a two-step molding technique [79] (Figure 3a). The two-level pillar array was designed to follow the actual size of gecko setae, and showed a sharply reduced *E_eff_* of ~ 26.3 kPa. However, adhesion strength of less than half of the single-level pillar array (~21 N/cm^2^) could be detected. Similar reduction in adhesion was also detected in the two-level polydimethylsiloxane (PDMS) pillars with different ARs, molded from a template prepared by two-step photolithography [80] (Figure 3b). The array of two-level PDMS micropillars has an *E_eff_* smaller than the single-level ones, by a factor of 3–7. However, due to the round edges of the second-level pillar and the partial misalignment of pillars, the adhesion of the two-level PDMS pillars was 10 times weaker. The reduced adhesion performances of these two examples strongly suggest the importance of the tip geometry of the secondary hierarchical structure. 

In order to gain better adhesion, the modified inking–printing–curing (IPC) technique was adapted to fabricate hierarchical self-similar micropillar arrays of PDMS and polyurethane (PU) [81,82]. By integrating the mushroom-shaped tip to the pillars in a self-similar two-level PU pillar array, the possibility to form effective contacts is much higher. The possibility of contact formation can be indicated by the indentation depth. The two-level array has an indentation depth of 441 μm, which is several times larger than the single-level array with the size of the first-level (indentation depth 138 μm) or the second-level structure (indentation depth 283 μm), and the flat control (indentation depth of 120 μm), showing high compliance to the counterpart surface. The two-level PU pillar array therefore showed ~10% and ~56% enhancements in adhesion, as compared to the single-level arrays; with the pillar size identical to that of the first- and second-level pillars, respectively (Figure 3c,d). Moreover, this hierarchical pillar array showed a stronger dependence on the loading force, as compared to the single-level pillar arrays (Figure 3d), suggesting again that a higher contact possibility could be achieved by reducing the *E_eff_* of the structure.

Due to easy handling in the nanometer scale [24], multi-branched anodic aluminum oxide (AAO) has been used to fabricate two-level hierarchical nanopillar arrays with various materials, including poly(methyl methacrylate) (PMMA) [83], polystyrene (PS) [84], poly(pentafluorophenyl acrylate) (poly(PFPA)) [85], Lexan polycarbonate (PC) [86], and so on. For example, two-level hierarchical Lexan PC nanopillar array with branched secondary nanopillars (~90 nm in diameter and ~850 nm in length), atop the primary nanopillars (~280 nm in diameter and ~5.5 μm in length), were obtained by capillary-assisted molding of multi-branched AAO template [86] (Figure 3e). The presence of the second-level nanopillars resulted in a shear adhesion force 1.5 times larger than those on a single-level pillar array. A rigid fluoropolymer (Teflon AF) with *E_inh_* of 1.5 GPa, comparable to β-keratin, was applied to fabricate high AR nanopillars with fluffy sheet-like terminals by using a AAO template combined with thermocapillarity-driven stresses [87]. Even when possessing the self-lubricating property, the two-level Teflon AF structure could reach a large shear adhesion strength of ~12 N/cm^2^, slightly larger than that of the gecko toe pad (10 N/cm^2^).

Similarly, techniques using the nickel oxide template [88], 3D direct laser writing [89], and imprinting [90] have also been involved in the fabrication of gecko-inspired hierarchical pillar arrays (Figure 3f,g). For example, an imprinting process was used to glue vertically-aligned carbon nanotube forests (CNTFs) onto the array of SU-8 micropillars, forming a two-level self-similar pillar array (Figure 3g). Although carbon nanotube (CNT) has an *E_inh_* of 4 GPa, the particularly large AR of the second-level CNTFs, and the two-level hierarchy, offer the structure an *E_eff_* of 1.6 MPa which is three orders of magnitude lower than the *E_inh_* of CNT. The small diameter (a few nanometers) and large density of CNTFs could gain millions of contact points on rough surfaces with an average surface roughness of R_a_ = 200 nm. The shear stress reached 185 N/cm^2^ which is over three times higher than that of the SU-8 pillar array and nearly one order of magnitude higher than that of CNTFs. After the initial shearing, the shear adhesion is reduced by 50% due to the permanent plastic deformation of the CNTFs along the shear direction.

**Figure 4:**
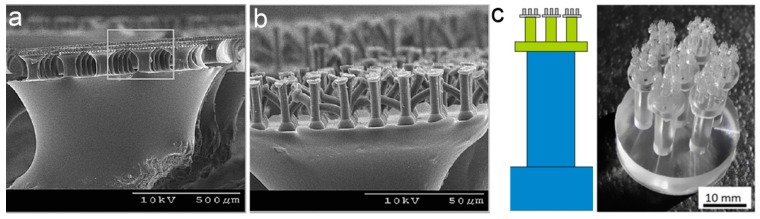
Three-level self-similar pillar structures. Scanning electron microscopy (SEM) images of (**a**) three-level polyurethane (PU) pillars with mushroom-shaped tips, and (**b**) the collapse phenomenon of the third-level. Reprinted with permission from [82]. Copyright (2009) American Chemical Society; (**c**) Schematic and image of the three-level polydimethylsiloxane (PDMS) macropillar adhesive. Reproduced with permission from [93].

Self-similar pillar arrays with three levels of hierarchy have also been successfully fabricated. However, the low packing density and structure defects hinder adhesion enhancement. For example, three-level PU (*E_inh_* ~ 3 MPa) pillars with a mushroom-shaped tip in each level (Figure 4a,b) were fabricated by the same method for the two-level pillars shown in Figure 3c [82]. The complexity of the fabrication process, and the slim structure, collapse part of the third-level PU pillars (hampered by the formation of proper contacts). To prevent the collapse of these fine pillars, stiff polypropylene (PP) with *E_inh_* of 1.5–2 GPa was applied to fabricate multi-level pillar arrays by the use of layered porous PC membranes with as different pore sizes as the mold [91,92]. While an ~12 and 25% adhesion enhancements were demonstrated in the one- and two-level pillar arrays (as compared to the flat reference, respectively), adhesion properties can hardly be further improved in the three-level structure. The *E_inh_* of PP is almost three orders of magnitude higher than PU; however, problems at the three-level PP structure remained. Instead of using a more rigid material, the increase in feature sizes may overcome the stability issue. The three-level hierarchical macropillar array at the millimeter and sub-millimeter scales was prepared by Arzt’s group [93] (Figure 4c). Once again, worse adhesions were detected. Though a hierarchical structure with three or even more levels possesses lower *E_eff_* and higher compliance with rough surfaces, it may be not the effective way to enhance the adhesion performance of a self-similar pillar array. Technically, it is also difficult to prepare a self-similar pillar array with a hierarchy of more than three levels, though the natural adhesives are even more complex hierarchical structures. 

#### 3.1.2. Structural Parameters of Pillar Arrays

**Figure 5:**
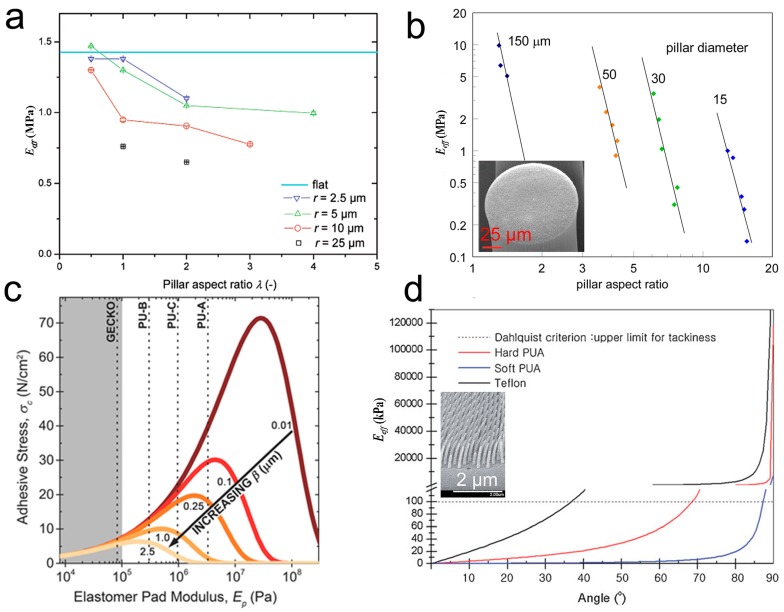
Manipulation of effective elastic modulus (*E_eff_*) by adjusting the structure parameters of pillar arrays. (**a**) Influence of the aspect ratio (AR) and pillar diameter on *E_eff_*. Reprinted with permission from [94]. Copyright (2007) American Chemical Society; (**b**) Dependence of *E_eff_* on the AR of micropillars, composed of a vertically aligned carbon nanotube (VA-CNT) array. The inset shows a scanning electron microscopy (SEM) image of the VA-CNT array. Reprinted with permission from [77]. Copyright (2012) American Chemical Society; (**c**) Adhesive stress capacity vs. elastomer pad modulus for varying roughness surfaces. Reproduced with permission from [101]; (**d**) Dependence of the *E_eff_* on the tilting angle of polyurethane acrylate (PUA) nanopillar array. The inset shows an SEM image of the slanted PUA nanopillar array. Reproduced with permission from [102].

The structural parameters of the pillars in each hierarchical level are essential to the adhesion performance of the self-similar hierarchical pillar array. Equation (3) provides a clear clue to manipulate the *E_eff_* of the pillar array. According to Equation (3), both the decrease in *d* and the increase in *l* can cause an increase in the AR, therefore reducing the *E_eff_* (which in turn contributes to the enhancement of adhesion). The fabrication of the pillar array with flat tips decreased the *E_inh_* of PDMS from ~1.4 to 0.6 MPa [94], and achieved a three-fold enhancement of pull-off force when the AR increased from 0.5 to 4 (Figure 5a). The carbon nanotube array is a typical example with extremely high AR: several angstroms to ~6 nm in diameter, and hundreds of micrometers in length [95]. The micropillar array (AR ~ 15), composed of a vertically aligned carbon nanotube (VA-CNT), has an *E_eff_* of ~0.2 MPa, which is 7 orders of magnitude smaller than the *E_inh_* of carbon nanotube (~1000 GPa) [77] (Figure 5b). Vertically aligned, multi-walled CNTs, with curly entangled ends and a high AR on a smooth surface, could reach almost 10 times stronger adhesion force than the gecko toe pad [96]. Pillars with a smaller diameter and larger length have the inherent ability to penetrate into the valley of rough asperities, acquiring more contact points for adhesion enhancement. Taking advantage of this effect, pillar dimensions must be chosen in relation to the roughness parameters of the contacting surface [97]. For a micropillar array, nanoroughness decreased adhesion strength, although the micropillar array retained higher adhesion strengths than the unpatterned controls [98]. The size matching between the pillar and the surface asperities can introduce geometric interlocking to the adhesion enhancement [97,99,100]. On the other hand, it has been also reported that adhesion drops dramatically when roughness approaches the size and spacing of the pillar features [39], similar to that found in gecko adhesion [34,35,36,37,38,39]. Furthermore, as substrate roughness increases, *E_eff_* of the adhesive pad should shift to a lower value to maintain the highest adhesion strength, but the adhesive stress capacity decreases (Figure 5c) [101].

Decreasing the tilting angle *θ* of the pillar is also an effective way to reduce *E_eff_* of a bioinspired pillar array, especially when a material with small *E_inh_* is used (Figure 5d) [102]. Moreover, a slanted pillar array offers great opportunity to gain anisotropy adhesion in directions following and against the tilting direction of the pillar [103,104]. An array of bended/tilted PUA Janus nanopillars, with one side coated with several nanometer-thick platinum, showed a shear adhesion force of ~31 N/cm^2^ in the tilting direction of the pillar, which was 7.5 times more than that in the opposite direction [105]. Interestingly, a recent report [100] indicates the sliding of the tilted pillar array along or against the tilting direction; the adhesion force first increased with the increase of roughness (due to feature matching of the two surfaces), and then decreased rapidly with the further increase of roughness. 

**Figure 6:**
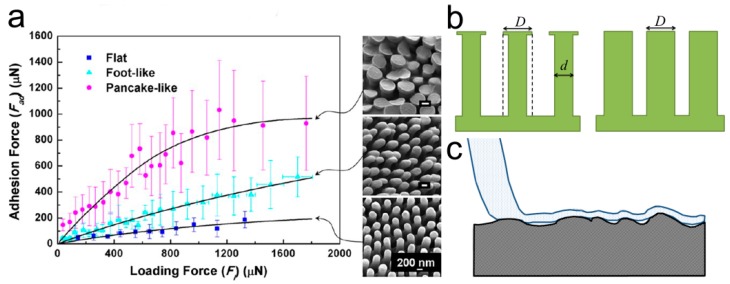
Adhesion performances of pillars with various tip geometries. (**a**) Influence of the tip geometry of polystyrene (PS) nanorods on adhesion forces. A scanning electron microscopy (SEM) image of the corresponding tip is shown in the right column. Reproduced with permission from [24]. Copyright (2012) American Chemical Society; (**b**) Schematic of a pillar array with mushroom-shaped and flat tips of diameter *D*; (**c**) Schematic diagram of a spatular tip contacting with a rough surface.

In addition to the structural parameters involved in Equation (3), the tip geometry of the pillar (flat, concave, mushroom-shaped, spherical, spatula-shaped, etc.) has a certain influence on the *E_eff_* of a patterned surface, altering the adhesion performance prominently [24,106,107] (Figure 6a). For instance, the pull-off force of pillars with mushroom-shaped tips can reach 30 times that of the flat controls [108]. A model of composite can be used to qualitatively estimate the *E_eff_* of the pillar with the mushroom-shaped tip (Figure 6b). The part beneath the overhang (*D* in diameter) is the second component (air) around the pillar stalk (d in diameter). According to Equation (1), the *E_eff_* of the array of the “composite pillar” is much smaller than the array of the pillar with the flat tip and a diameter of *D*. The larger diameter *D* of the overhang can, on the one hand, further reduce *E_eff_*, and on the other hand can form a larger contact area with the counterpart surface. Moreover, the mushroom-shaped tip can insert certain vacuum pressure during the pull-off, enlarging the adhesion forces [109,110]. If the overhang is not symmetric, like the spatula-shaped tip, anisotropy adhesion can be obtained [111,112,113]. The pillar with the stepped mushroom-shaped tip offers different contact areas in opposite directions, and the ratio of adhesion strength obtained in different directions exceeds 20 [71]. 

The tip shapes of the pillar have a strong impact on the adhesion strength of surfaces with various roughnesses. The mushroom-shaped terminal of the tarsal adhesive setae of the Colorado potato beetle (*Leptinotarsa decemlineata*) has been proved to be suitable for smooth surface and long-term adhesion processes, while the spatular tip on the setae applies to the short-term process and locomotion on rough surfaces [36]. The thin spatular tip can adapt itself to rough surfaces increasing the effective contact points (Figure 6c) [19]. Pillars with spatular tips were found to increase adhesive forces by 10 times on sinusoidal surfaces, with amplitudes much larger than the nanoscale features and 2.5 times on smooth surfaces [39]. Moreover, the spatular tip was found to enhance adhesion on surfaces with a certain roughness more effectively than the pillars with spherical, flat, torus [114], and hemispherical tips [39]. 

### 3.2. Lamella–Pillar Hybrid Structure

**Figure 7:**
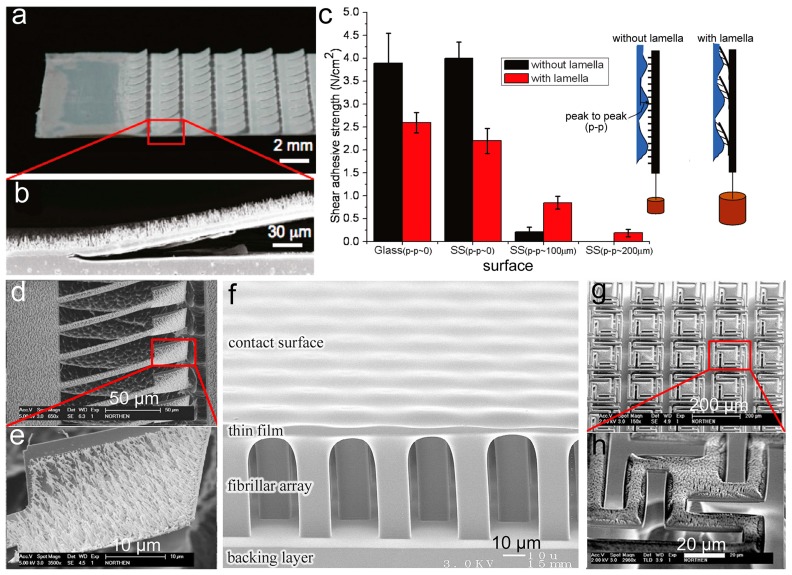
Different lamella–pillar hybrid (LPH) structures. (**a**,**b**) High-density polyethylene (HDPE) nanopillar array supported by lamellar flaps (LPH-1); (**c**) Comparison of shear adhesion strength between the LPH structure (with lamella) and the single pillar array (without lamella), contacted with surfaces of different roughnesses (SS: Stainless steel). (**a**–**c**) Reprinted with permission from [115]. Copyright (2009) American Chemical Society; (**d**,**e**) Nickel paddle coated with a Photoresist nanorod array. Reproduced with permission from [116]; (**f**) Thin film-terminated fibrillar arrays (LPH-2). Reproduced with permission from [119]; (**g**,**h**) Photoresist nanorod array on top of a SiO_2_ platform supported by a single-crystal silicon pillar. Reproduced with permission from [121].

The lamella–pillar hybrid (LPH) structure is a structure combining pillars and thin membranes, mimicking the hierarchical structure (setae on lamella) found on the gecko toe pad (as shown in Figure 2b). The first kind of LPH (LPH-1) is composed of a pillar array supported by a thin film, similar to the gecko adhesive. The LPH-1 structure, composed of a high-density polyethylene (HDPE) nanopillar array (600 nm in diameter and 18 μm in height) on the HDPE lamellar flaps (15 μm in thickness, 0.8 mm in width and 1.3 mm in length), was fabricated by using heated rollers and PC templates (Figure 7a,b) [115]. The micrometer compliance provided by the nanopillar arrays, together with the sub-millimeter compliance, allowed the lamellar flaps to offer the HDPE LPH-1 structure ~160 times larger compliance than that of the nanopillar array without lamellar flaps. The enormously enhanced compliance thus allowed the hybrid structure to acquire five times greater shear strength on the rough surface with peak-to-peak = 100 μm than the nanopillar arrays without the lamellar flaps, even though the gaps between the lamellar flaps reduced 58% of the apparent contact area (Figure 7c). However, the shear strength of the HDPE LPH-1 structure on smooth surfaces (glass and stainless steel) can only reach 63% of the nanopillar arrays. One step further, a similar LPH-1 structure (of aligned vertical photoresist nanorod array atop nickel paddle) was prepared to gain reversible adhesions by controlling the rotation of paddles with a magnetic field [116] (Figure 7d,e).

The second kind of LPH (LPH-2) has a thin film on top of the pillars (Figure 7f) [117,118]. In contrast to LPH-1, the contact with a counterpart surface occurs on the thin film side of LPH-2. Although LPH-2 is the upside-down structure of the gecko adhesive, the PDMS LPH-2 showed a pull-off force larger than the flat control by a factor of 1.5–3.5. The small *E_eff_* of the supporting layer (in this case the pillar array) allows the film on top to easily deform, maximizing the contact area and the adhesion force [119]. Moreover, the LPH-2 structure has an extra advantage with the terminal film able to keep the pillars from collapsing or bucking, contributing to the high stability of LPH-2. The pillar array could be further introduced onto the thin film of LPH-2, forming a pillar–film–pillar sandwich structure. For instance, microscaled wedge-shaped PDMS pillars were added to the PDMS film atop an array of slanted PDMS stalks by a molding process [120]. The *E_eff_* of the sandwich structure was measured to be 15–25 kPa, only 2–4% of the *E_inh_* of PDMS. Thus, the sandwich structure could adapt to rough surfaces (granite with an RMS roughness of 21 μm and even roughly sanded pine) much easier, improving adhesion by a factor of five compared to the wedge-shaped pillars alone. LPH-2 structures could stack together to form a multilayer structure, further enhancing adhesion.

The thin film in a pillar–film–pillar sandwich structure could be designed into certain patterns. For instance, a photoresist nanorod array (2 μm in length and 50–200 nm in diameter) was assembled onto a SiO_2_ platform (20–150 μm) supported by a single high aspect ratio pillar made of single-crystal silicon, forming an array of pillar–film–pillar structures [121] (Figure 7g,h). Although formed by hard and brittle materials, this hybrid structure was still more compliant than the single nanorod array when contacted with uneven aluminum surfaces.

### 3.3. Porous Structure

**Figure 8:**
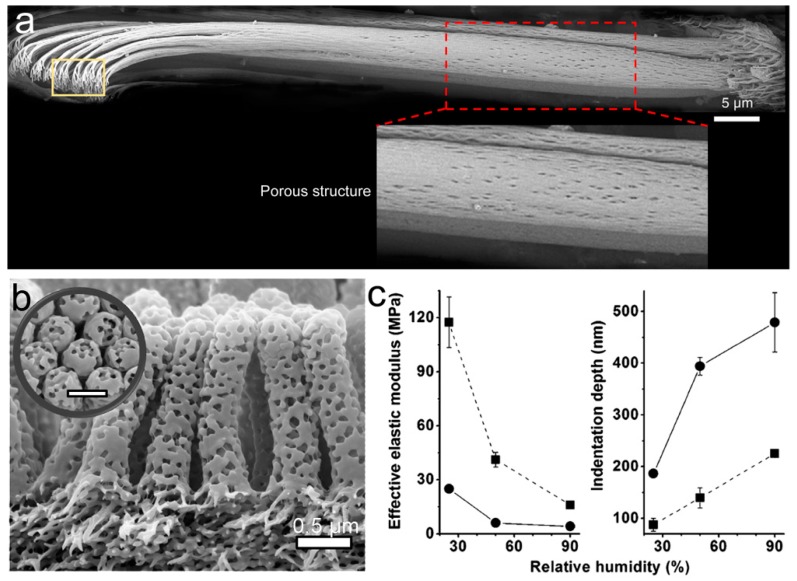
Biological and bioinspired porous pillars for adhesion. (**a**) Scanning electron microscopy (SEM) image of a single dried seta of tokay gecko (some pores can be found on the stalk). Reproduced with permission from [10]; (**b**) SEM image of the porous fibrillar polystyrene-*b*-poly(2-vinyl pyridine) (PS-*b*-P2VP) adhesive. Reproduced with permission from [124]; (**c**) Effect of the relative humidity on the *E_eff_* and indentation depth. Reprinted with permission from [123]. Copyright (2013) American Chemical Society.

The porous structure is a hierarchical structure with random pores or channels embedded in a matrix. As show in Figure 8a, dense pores were found in a dry seta of the tokay gecko [10], which could lower the *E_eff_* of the setae and increase compliance. The *E_eff_* of a porous material can be given as follows [122]:

(4) $$E_{eff}=E_{0}\times e^{-\left(bp+cp^{2}\right)}$$

where *b* and *c* are the material constants, and *p* is the porosity. From Equation (4), the *E_eff_* of the porous structure could be much smaller than the *E_inh_* of the corresponding material due to the inverse relationship between *E_eff_* and porosity. Block copolymer polystyrene-*b*-poly(2-vinyl pyridine) (PS-*b*-P2VP), with an appropriate block ratio, was used to fabricate a bio-inspired porous fibrillar adhesive (Figure 8b) [123]. The solid fibrillar array (~300 nm in diameter) was replicated from the self-ordered AAO template by capillary wetting. The following swelling of the poly(2-vinyl pyridine) (P2VP) block in its good solvent ethanol converted the solid fibril into a porous fibril. The nanopores (mean diameter of ~98 nm) in the fibrillar array reduced the *E_eff_* of the solid fibrillar array from ~41.2 to ~6.0 MPa. Moreover, the *E_eff_* of the porous fibrillar array could be further reduced by exposing the array to high humidity, as the polar component P2VP could absorb a certain amount of water and become softer (Figure 8c). Increasing the relative humidity from 2 to 90% could, therefore, enhance adhesion by a factor of ~6. Additionally, the pores in the PS-*b*-P2VP fibrillar array were used to deliver mineral oil, which mimics the adhesive secretion of some insects, to the contact areas rendering wet adhesion. The synergistic effect of capillarity and humidity-induced decrease in *E_eff_* appears to improve the adhesion properties of this porous structure by two orders of magnitude [124].

**Figure 9:**
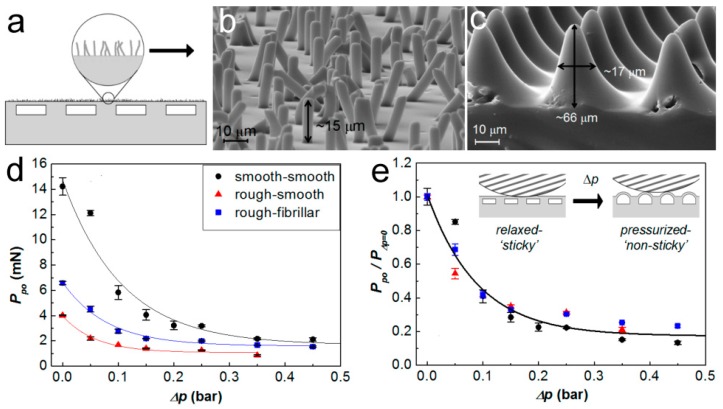
Polydimethylsiloxane (PDMS) film embedded with microchannels for adhesion. (**a**) Schematic of the channel structure with textured outer surfaces and scanning electron microscopy (SEM) images of (**b**) fibrillar and (**c**) conical pillars. (**d**) Dependence of the pull-off force (*P*_po_) and (**e**) the *P*_po_ normalized to the preload (*P*_Δpo=0_) on the increase of differential pressure in the microchannels (Δ*p*). Reproduced with permission from [129].

Embedded microchannels were also used to reduce the local *E_eff_* and therefore the adhesion properties of the corresponding structure [125]. The aligned microchannels and the spaces between channels thus create a pattern of *E_eff_* on the surface. Interestingly, this kind of *E_eff_* pattern could even be sensed by living cells [126]. By adjusting the pressure inside the evenly embedded microchannels within a PDMS film with silicone oils of different viscosity (5 to 50,000 mPa∙s), controllable adhesion was achieved [127]. It should be noted that the top surface of the PDMS film is unpatterned and smooth. Furthermore, the pressure inside the microchannels could regulate the deformation of the top surface for adhesion switching. By the synthetic control of liquid pressure and other structural parameters, the maximum adhesion strength could be ~30 times higher than that of the unstructured PDMS film. Array of fibrils or conical pillars was also combined onto the surface of the channel-embedding PDMS film (Figure 9a–e) [128,129]. This kind of structure showed better adhesion on a rough surface (RMS roughness of 3.7 µm—the “rough-fibrillar” curve in Figure 8d) than on a flat surface (the “rough-smooth” curve in Figure 8d); even the latter one performed much better on a smooth surface (RMS roughness: 19 nm—the “smooth-smooth” curve in Figure 8d). Moreover, the adhesion efficiency (pull-off force (*P*_po_) divided by the preload (*P*_Δpo=0_)) was reduced following the increase of pressure in the microchannels (Δ*p*) (Figure 8e).

## 4. Conclusions

Inspired by the structured adhesives in nature, various artificial adhesives have been developed to gain controllable adhesions. Based on structural features, we summarized the artificial adhesives as belonging to three groups: self-similar hierarchical pillars, the lamella–pillar hybrid structure, and the porous structure. Although these structures differ greatly from each other, they can effectively reduce the *E_eff_* of the material, contributing to compliance enhancement. The self-similar hierarchical pillar has the highest similarity to the gecko setae; however, it suffers from the difficulties of manufacturing and low structure stability. The LPH structure has the best stability, and its adhesion could be further enhanced by optimizing the structural parameters. The porous structure for bioinspired adhesion is still at its early stage, and needs further development; it has the advantage of being able to mimic the adhesive secretion of some animals and aims at wet adhesion mimicking. The combination of structural elements may pave the way to reach or even surpass the abilities of biological structured adhesives. Hierarchical structures have a much higher possibility of forming reliable contacts on natural rough surfaces with various wavelengths and amplitudes, aiming at final applications in the real world.
